# Electrochemical Sensing Platform Based on Carbon Dots for the Simultaneous Determination of Theophylline and Caffeine in Tea

**DOI:** 10.3390/s23187731

**Published:** 2023-09-07

**Authors:** Paola Di Matteo, Alessandro Trani, Martina Bortolami, Marta Feroci, Rita Petrucci, Antonella Curulli

**Affiliations:** 1 Department of Basic and Applied Sciences for Engineering, Sapienza University of Rome, 00161 Rome, Italy; p.dimatteo@uniroma1.it (P.D.M.); martina.bortolami@uniroma1.it (M.B.); marta.feroci@uniroma1.it (M.F.); 2 Consiglio Nazionale Delle Ricerche, Istituto per lo Studio dei Materiali Nanostrutturati, Unità Operativa di Supporto, Sapienza, 00161 Rome, Italy; alessandro.trani@ismn.cnr.it

**Keywords:** carbon dots-modified electrode, screen-printed carbon electrode, glassy carbon electrode, caffeine, theophylline, tea, drug, HPLC-ESI-MS

## Abstract

A simple and selective method for the determination of caffeine (CAF) and theophylline (THEO) has been developed for a glassy carbon electrode (GCE) modified with a composite including carbon dots (CDs) and chitosan (CS). To our knowledge, there are no previous studies that analyze a CDs-modified GCE for the presence of CAF and THEO. The electrochemical behavior of a GCE modified with a CDs-CS composite was studied in acidic medium by cyclic voltammetry (CV) and differential pulse voltammetry (DPV). Considering the sensor analytical parameters, the same linear concentrations range was found for CAF and THEO ranging from 1 × 10^−5^ to 5 × 10^−3^ mol L^−1^ with the same detection limit (LOD) of 1 × 10^−6^ mol L^−1^. The reproducibility and repeatability data were satisfactory in terms of RSD%. Moreover, the storage stability was evaluated, evidencing good results whatever the experimental conditions used. The developed sensor was applied for the simultaneous determination of CAF and THEO in tea and drug, and results were compared with those obtained with HPLC-ESI-MS in SIR mode as an independent method optimized on purpose. The electrochemical sensor presents the undoubled advantages in terms of cheapness, portability, and ease of use, since it does not require skilled personnel.

## 1. Introduction

Tea is among the most consumed natural beverages worldwide. Its composition and taste can differ substantially depending on the processing treatments. In fact, based on the fermentation degree, tea can be classified as unfermented tea, mainly preserving the fresh leaves composition, yellow tea, fermented for 10–20%, and black tea, fermented for 80–90% [1]. In addition to the appreciated organoleptic properties, antioxidant and anti-inflammatory effects as well as also reduced risk of cancer and cardiovascular disease have been associated with the consumption of tea [1,2]. The beneficial effects have been attributed mainly to the tea phenolic fraction, which is characterized by catechin and catechin derivatives [3], and to the bioactive compounds caffeine (CAF) and theophylline (THEO) that are alkaloids belonging to the methylxanthines class [4,5]. 

**Scheme 1:**
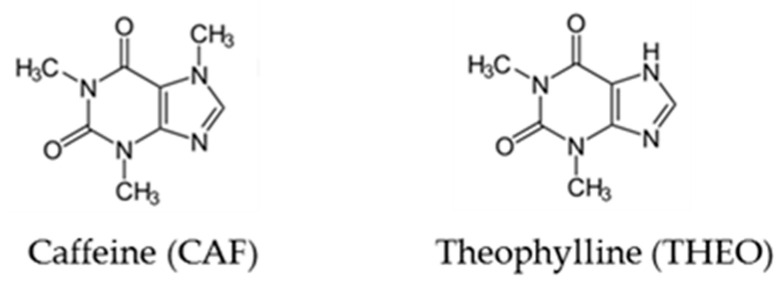
Chemical structures of CAF and THEO.

CAF (1,3,7-trimethylxanthine) and THEO (1,3-dimethylxanthine, structures in the Scheme 1) evidence particular pharmacological properties [4], whose beneficial effects depend on dose [6,7].

It is well known that CAF enhances physical resistance and mental focus as well as alleviates fatigue and headache, but an excessive use can induce anxiety, blood pressure increase, and cardiovascular diseases [4,8].

The pharmacological effects of THEO are exploited in drugs for asthma and chronic obstructive pulmonary disease [4,9]. In particular, it has been used worldwide for years to treat asthma, with a therapeutic window of 10–20 μg mL^−1^, and neonatal apnea, with a dose within 6–11 μg mL^−1^, since it is low cost and largely available [7]. It is noteworthy that while mild effects such as nausea and headache can be observed below 10 μg mL^−1^, more severe collateral effects such as tremors, agitation, insomnia, cardiac arrhythmias and seizures are common at concentrations higher than 20 μg mL^−1^. Thus, the development of accurate, fast and simple methods to monitor CAF and THEO in real samples, as beverages or drugs, is still an interesting topic.

Nowadays, different techniques are available for the quantitation of CAF and THEO based on high-performance liquid chromatography (HPLC) or gas chromatography (GC) coupled with mass spectrometry (MS) or fluorescence (FLD) detectors [10,11,12]. These techniques provide robust, sensitive and highly selective methods, generally requiring time-demanding sample pre-treatment like extraction, pre-concentration and derivatization, along with expensive instrumentation and skilled personnel. 

Otherwise, the electrochemical techniques need easy procedures, do not require too expensive equipment, and offer the possibility of miniaturization and predisposition for real-time and on-site analysis. In addition, the performance of electrochemical sensors can be improved by using nanomaterials, so nanomaterial-modified electrodes can be considered innovative diagnostic tools for monitoring and determining specific analytes [13]. 

Nowadays, carbon dots (CDs) seem to represent an interesting alternative with respect to the “older generation” of conventional carbon nanostructures [14]. They are assumed as zero-dimensional (0D) carbon nanomaterials and have been extensively used in the electrochemical (bio)sensing area [15,16], since they revealed low toxicity, high stability and biocompatibility. CDs are quasi-spherical carbon nanoparticles with an amorphous carbonized carbon core and different functional groups on their surface, depending on both the synthetic pathway and the starting materials [17]. It is noteworthy that their electrochemical properties may be considered similar to those of graphene (G) [16,18].

To the best of our knowledge, no study on the electrochemical behavior of CAF and THEO on a glassy carbon electrode (GCE) and a screen-printed carbon electrode (SPCE) both modified with CDs has been reported, even if many examples of sensing and biosensing platforms including CDs have been already reported in the literature [14,16].

In this contest, the present work aimed to investigate the potentiality of GCE and SPCE modified with electrosynthesized CDs for the simultaneous determination of CAF and THEO. Since CAF and THEO are both present in tea, commercial teas were chosen as real matrices to test the resulting optimized sensor. Moreover, due to the low content of THEO in teas with respect to CAF, as generally reported [19], a commercial drug containing THEO was analyzed, too, to evaluate the suitability of the presented method for the determination of higher amounts of THEO in a complex matrix. 

## 2. Materials and Methods

### 2.1. Chemicals and Solvents

All chemicals were of analytical grade, purchased from Merck (Rahway, NJ, USA)/Italy-Sigma-Aldrich (Milano, Italy) and used as received. In detail, these included the following: caffeine (CAF), theophylline (THEO), perchloric acid (HClO_4_), 70%, acetic acid (AA), potassium hydroxide (KOH) ≥ 85% pellets, sodium hydroxide (NaOH), potassium ferricyanide (K_3_[Fe(CN)_6_]), 5-caffeoylquinic acid (CQA), ferulic acid (FA), catechin (C), epicatechin (EC), and formic acid (FA). Ethanol (EtOH) was purchased from Merck/Italy-Sigma-Aldrich; HPLC-grade acetonitrile and methanol were purchased from Carlo Erba (Milano, Italy); HPLC-grade water was freshly prepared by the Milli-Q purification system (Millipore, Vimodrone, Italy). Medium-molecular-weight chitosan (CS, 5800 g mol^−1^), composed of β-(1-4)-linked d-glucosamine and *N*-acetyl-d-glucosamine with a degree of deacetylation of 75–85% was purchased from Merck/Italy-Sigma-Aldrich.

### 2.2. Tea and Drug Samples

Three commercial teas in filter bags were purchased from the local supermarket: Black Tea Darjeeling Coop (Coop Italia S.c.a.r.l. www.e-coop.it (accessed on 23 July 2023), Casalecchio di Reno, Italy), Twinings Agrumance Tea (Twining Crosfield and Company Ltd., London, UK) and Decaffeinated Earl Grey Green Tea (Everton Tea India Pvt. Ltd., Sri City, India). Each tea infusion was prepared by leaving one bag in 250 mL of incipient boiling ultrapure water for 3 min according to the recommended procedure. After removing the bag, tea infusion was left until room temperature was reached, filtered 0.22 μm and used for the analysis.

THEO-DUR 300 mg, RECORDATI Industria Chimica e Farmaceutica S.p.A.—Milano, an extended release drug containing THEO, is the only formulation available in Italy, and it was purchased from a local drugstore under prescription. One tablet (0.6475 g) was ground, dissolved in 300 mL ultrapure water and left under stirring at room temperature for 30 min; the resulting solution was filtered twice: 0.45 μm followed by 0.22 μm to remove the insoluble excipients, and the filtered solution was used for the analysis.

### 2.3. Carbon Dots Electrosynthesis and Characterization

Carbon dots (CDs) were electrochemically synthesized using EtOH as a carbon source as previously reported [17,20] by an Amel Model 552 potentiostat equipped with an Amel Model 731 integrator. Briefly, 1 mL of water containing 110 mg (2.75 mmol) of NaOH was added to 10 mL of EtOH, and the resulting solution was electrolyzed at constant anodic potential (E_ox_ = +3 V, vs. SCE) for 5 h under stirring, using a Pt working electrode, a Pt counter electrode and a saturated calomel reference electrode (SCE). Ethanol (10 mL) was added to the electrolyzed solution and left overnight to salt out NaOH. The solution was then centrifuged for 5 min at 5000 rpm by an ALC Centrifugette 4206, and the supernatant was first reduced to 3 mL under vacuum, then supplemented with 3 mL of water and finally dialyzed against ultrapure water (600 mL) through a dialysis membrane (MWCO 0.1–0.5 kD) for 48 h, changing the dialysis water after 24 h. The dialyzed solution was filtered at 0.2 μm, concentrated under vacuum and dried by the Smart Evaporator.

The resulting orange solid was analyzed by Scanning Electrode Microscopy (SEM), which was performed with a High-Resolution Field Emission Scanning Electron Microscope (HR-FESEM) AURIGA Zeiss; the images were taken under 200 nm to verify the average size of the electrogenerated CDs. The IR spectrum was recorded by a Bruker LUMOS II FTIR spectrophotometer in ATR mode, and fluorescence measurements were performed with a Fluoromax-3 Horiba Jobin–Yvon fluorometer (T = 25 °C), whose data were corrected by a built-in program to counterbalance the decay in sensitivity in the near-infrared region and divided by the corrected reference detector. 

### 2.4. Electrochemical Measurements

Electrochemical measurements were carried out by an Autolab PGSTAT12 potentiostat/galvanostat (Metrhom Autolab BV, Utrecht, The Netherlands) by using a conventional two-compartment three-electrode cell, a glassy carbon electrode (GCE, 2 mm in diameter), purchased by Metrohm Autolab BV (Utrecht, The Netherlands), as the working electrode, a Pt counter electrode and an Ag/AgCl reference electrode. Moreover, screen-printed carbon electrodes (SPCEs), purchased from Metrohm Autolab BV (Utrecht, The Netherlands), were used. Each SPCE includes a traditional three-electrode system printed on the same strip. In this work, SPCE 110 and SPCE 150 were used, both including a carbon working electrode (4 mm in diameter) and a silver reference electrode. SPCE 150 has a Pt counter electrode, while SPCE 110 has carbon as the counter electrode.

The CDs-CS composite was prepared as follows: 0.5 mg of CDs powder was suspended in 1 mL of distilled water and sonicated for 5 min; CS was dissolved in aqueous acetic acid (0.1 mol L^−1^) until obtaining a 1.0 wt% solution and sonicated for 20 min; the CDs and CS solutions were then mixed in the ratio 1:1, *v*:*v*.

Prior to modification, GCE was polished with 10 nm aluminum oxide powder, successively immersed in water and EtOH for ultrasonic cleaning, and subjected at polishing steps.

GCE and SPCEs were both modified through two different approaches, namely drop casting and electrodeposition.

In the drop-casting procedure, a proper volume (2 μL) of the CDs-CS solution was casted on the electrode surface and after rinsing with distilled water, the electrode was air dried naturally at room temperature for 1 h to obtain the CDs-CS-modified electrode.

In the electrodeposition method, the electrode was scanned at 50 mV s^−1^ for 50 cycles by using 60 μL of the CDs-CS solution, from −0.8 to +0.8 V, modifying a method already reported in the literature [21]. After rinsing with distilled water, the resulting CDs-CS-modified electrode was air dried naturally for 20 min.

The efficiency of the electrode surface modification was evaluated by cyclic voltammetry (CV) at 0.020 V s^−1^, using 1 × 10^−2^ mol L^−1^ K_3_[Fe(CN)_6_] as the redox probe, comparing peak potential and peak current intensity data obtained at bare and the corresponding modified electrode. The electrochemical behavior of the redox probe at the bare and modified electrodes was studied by CV in the scan rate range 0.020–0.500 V s^−1^.

The electrochemical behavior of CAF and THEO at bare and modified electrodes was studied by CV at 0.020 V s^−1^, and by differential pulse voltammetry (DPV) in the positive potential range 0.30–1.80 V, using the following optimized parameters: step potential 0.004 V, modulation amplitude 0.025 V, modulation time 0.05 s, and scan rate 0.02 V s^−1^. Measurements were carried out on ultrapure water solutions containing CAF and THEO; in turn, 5.0 × 10^−3^ mol L^−1^; HClO_4_ was used as a supporting electrolyte in the pH range 0.4–7.0. 

All the measurements were carried out at room temperature and without deareating as oxygen does not interfere in the anodic potential window.

Data acquisition, data handling, and instrument control were performed by the Autolab NOVA 1.10 software system.

### 2.5. Electrochemical Analysis

CDs-CS/GCE was used to construct the calibration curves for CAF and THEO in the optimized 0.4 mol L^−1^ HClO_4_ solution (pH = 0.4), according to the standard addition method, reporting the anodic peak current (*I*_ap_) mean value ± standard deviation (SD) of six repeated DPV measurements for each point of the curve, in the concentration range 1 × 10^−5^–5 × 10^−3^ mol L^−1^. The calibration curves were analyzed by linear least-square regression in Origin Pro 8.1 (Origin Lab Corporation, USA). The limit of detection (LOD) was obtained by using the equations LOD = 3sx/y/b where sx/y and b were the estimated standard deviation and the slope of the analytical calibration function with a 95% confidence level [22]. Precision for each modified electrode was evaluated using seven electrodes (*n* = 7).

CDs-CS/GCE was used to quantitate CAF and THEO in real matrices, namely three teas and one drug, as follows: an aliquot of tea infusion (5 mL) prepared as described in Section 2.2. was added to HClO_4_ 70% (*w*/*w*) until the optimized pH value of 0.4 was reached and analyzed by DPV reporting the anodic peak current (*I*_ap_) mean value ± standard deviation (SD) of three repeated measurements. A similar procedure was applied to the drug solution (5 mL) prepared as described in Section 2.2. 

### 2.6. HPLC-ESI-MS in SIR Mode Analysis

Measurements were carried out by a 1525μ HPLC Waters (Milford, MA, USA) coupled with a Quattro Micro Tandem Mass with an electrospray ionization (ESI) source Waters (Micromass, Manchester, UK); a Waters XBridge C18 (150 × 2.1 mm i.d.) 5 μm analytical column was used for the separation; A (water/FA 0.02%) and B (acetonitrile/FA 0.02%) were used as mobile phase, flow rate 0.20 mL min^−1^. The chromatographic separation was optimized as follows: 0 min, 5% B; 0–10 min, 30% B; 10–12 min, 30% B; 12–22 min, 80% B, to completely elute the other analytes contained in the real matrices; 22–23 min, 5% B; 23–43 min, 5% B, to equilibrate the column before a new run. Mass spectral data were acquired in positive ionization mode (ES+) by using the Selected Ion Recording (SIR) technique [23], with the following optimized source parameters: capillary voltage 3000 V, cone voltage 20 V, source temperature 120 °C, desolvation temperature 350 °C, cone gas flow 40 L h^−1^, desolvation gas flow 600 L h^−1^. The monoisotopic values 195 *m*/*z* and 181 *m*/*z* for the protonated ion [M+H]^+^ of CAF and THEO, respectively, were selected in two independent acquisition channels.

Data acquisition, data handling, and instruments control were performed by MassLynx Software 4.1 v (Data Handling System for Windows, Micromass, UK).

The calibration curves for the quantitation of CAF and THEO were obtained as follows: 1 mg mL^−1^ of the standard compound was dissolved in methanol; working solutions at the final concentrations of 50, 100, 300, 500, 700, 1000 μg L^−1^ were prepared by appropriate dilution with the mobile phase (A:B, 95:5, *v*:*v*) and injected in triplicate (20 μL). The calibration curves were calculated with equal-weighted least-squares linear regression analysis of the SIR peak area against the standard nominal concentration. Limit of detection (LOD) and quantitation (LOQ) were obtained as LOD = 3Sa/b and LOQ = 10Sa/b, respectively, where Sa and b are the estimated standard deviation and the slope of the analytical calibration function with a 95% confidence level, respectively [23].

Tea and drug samples, prepared as described in Section 2.2., were appropriately diluted with the mobile phase (A:B, 95:5, *v*:*v*) and injected in triplicate for the analysis (20 μL). Results are reported as mean values ± standard deviation (SD). 

### 2.7. Statistical Analysis

Electrochemical and chromatographic data were analyzed by using the one-way analysis of variance (ANOVA). The significance of differences (*p* < 0.05) among samples was determined by the Tukey test [24,25].

The matrix effect (ME) was evaluated by HPLC-ESI-MS comparing the matrix-matching calibration curve (50, 100 and 200 μg L^−1^, for tea samples; 1000, 1500 and 3000 μg L^−1^, for drug sample) with the corresponding calibration curve of the standard CAF and THEO, according to the literature [26].

## 3. Results and Discussion

The present study aimed to investigate the possibility of developing an electrochemical sensing platform for the simultaneous determination of two analytes, caffeine and theophylline, by using an electrosynthesized carbon dots/chitosan composite to modify conventional electrodes. For this purpose, a glassy carbon electrode (GCE) and screen-printed carbon electrode (SPCE) were chosen as the starting electrode material, the second ones presenting the advantage, if well performing, of being suitable as a portable tool for on-site analysis. The modified electrodes were used for all the electrochemical measurements, that were carried out in acidic medium at different pH values, by following the anodic oxidation of CAF and THEO, simultaneously and individually, by CV and DPV. After comparing the obtained results, the best-performing sensor and the optimized operative conditions were tested for the quantitation of CAF and THEO in real matrices. Detailed results are discussed below. 

### 3.1. CDs Electrosynthesis and Characterization

Carbon dots (CDs) were electrochemically obtained (Section 2.3) from a solution of water, EtOH and NaOH, in which ethanol was the carbon source. Electrolysis was carried out under potentiostatic conditions (E_ox_ = +3 V, vs. SCE) using platinum electrodes, and the obtained nanoparticles were purified by centrifugation and dialysis. The so obtained material was characterized by SEM, infrared and fluorescence analysis (Section 2.3) and results were compared with the literature [15,20,27,28,29].

**Figure 1:**
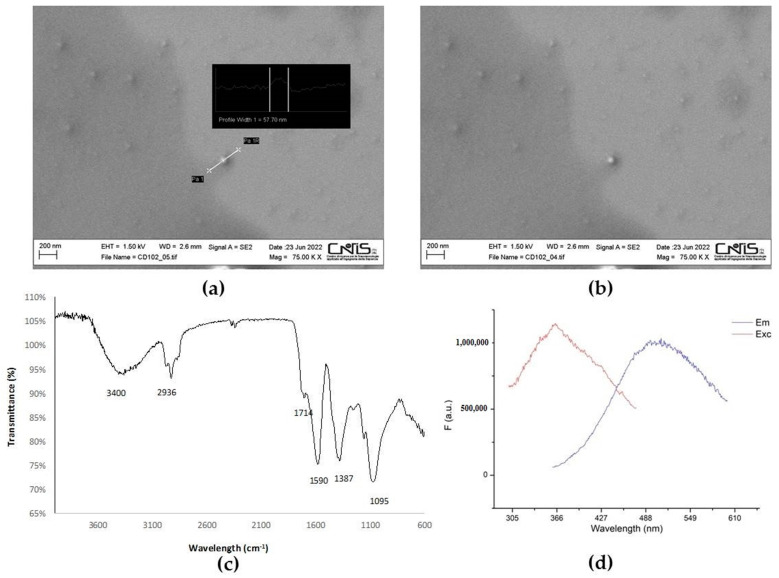
Characterization of electrochemically synthesized CDs: (**a**,**b**), scanning electron microscopy (SEM) images; (**c**) IR spectrum (ATR mode); (**d**) fluorescence excitation (red line) and emission (blue line) spectra (CDs, 2 mg/mL).

SEM images (Figure 1a,b) showed nearly spherical nanoparticles with dimensions in the range of 40–70 nm [15,20]. 

IR spectrum (Figure 1c) showed the presence on the surface of O-H groups (O-H stretching vibrations centered at 3400 cm^−1^, O-H in-plane bending near 1387 cm^−1^, C-O stretching near 1095 cm^−1^), C-H groups (stretching near 2936 cm^−1^ and bending near 1387 cm^−1^), and unsaturated carbons (C=C stretching at 1590 cm^−1^ and in-plane bending around 1095 cm^−1^). A weak peak at 1714 cm^−1^ could be due to C=O stretching, possibly acids or esters. All these functional groups are coherent with the anodic oxidation of ethanol under basic conditions, and they are in good agreement with literature [20,27]. Polar groups evidenced by IR spectrum are consistent with the very good solubility evidenced in water.

Moreover, these nanoparticles showed, as expected, a good fluorescent behavior, with a maximum of absorbance around 365 nm and a corresponding emission maximum around 490 nm (Figure 1d), which is in good agreement with the literature [20,28,29].

### 3.2. Electrochemical Characterization of CDs-Modified Electrodes 

The electrosynthesized CDs were used as nanocarbon material and mixed with chitosan to form the nanocomposite for the electrode surface modification. In fact, it is well known that CS is a functional material showing good adhesion, film-forming ability, and biocompatibility, so it is considered a good material to develop sensing platforms [30,31]. On the other hand, CS is a non-conducting biopolymer; for this reason, it is generally combined with nanomaterials to improve its conductivity [22,32,33]. Moreover, satisfactory results for the quantitation of CAF in beverages were previously obtained by using a gold nanoparticles–chitosan (AuNPs-CS)-modified electrode [22], whose peculiar performance was explained by the synergistic action of AuNPs and CS: AuNPs guarantee a more efficient electron transfer, whereas the CS functional groups facilitate the interaction between CAF and the electrode surface. In the CDs-CS-modified electrodes herein investigated, CDs have a role similar to AuNPs, and CS ensures an efficient interaction between the analyte and the electrode through the functional groups present in its structure.

All the electrodes, GCE and SPCEs, were modified following two deposition methods, i.e., drop casting and electrodeposition, and the efficacy of the surface modification was evaluated by means of CV, using 1 × 10^−2^ mol L^−1^ K_3_[Fe(CN)_6_] as a redox probe. 

Although a peak current intensity increase was generally observed, two different behaviors were evidenced.

The higher increase was observed by drop-casting modification (52.63% vs. 4.68%) in the case of GCE, while the best modification was obtained by electrodeposition in the case of SPCEs 110 and 150 (28.70% vs. 10.74% for SPCE 110 and 26.90% vs. 8.35% for SPCE 150). A possible explanation consists of the fact that the electrochemical activation of the amorphous carbon surface by means of CV is required for an effective CDs deposition onto the SPCEs surface. Conversely, the best efficacy of drop-casting modification observed for GCE might be due to the different properties of the glassy carbon material [33,34]. CV data relative to bare electrodes and the corresponding best-performing modified electrodes are resumed in Table 1.

A diffusion-controlled process was observed for the redox probe at all the electrodes, as supported by linear plots of *I*_ap_ vs. v^1/2^ (where *I*_ap_ is the peak current intensity and v is the scan rate in the range 0.02–0.50 V s^−1^), which is in agreement with literature findings for nanostructures [34,35]. 

An amplification of the electrochemical response was observed for the redox probe at all the modified electrodes with respect to the corresponding bare electrode, ranging from 52.63 to 26.90% (see Table 1). The current increase might be explained by the higher surface area of nanostructured electrodes [34], which was calculated by CV in 1.0 × 10^−2^ mol L^−1^ K_3_[Fe(CN)_6_] according to the Randles−Sevcik equation [35] (Table 1). 

**Figure 2:**
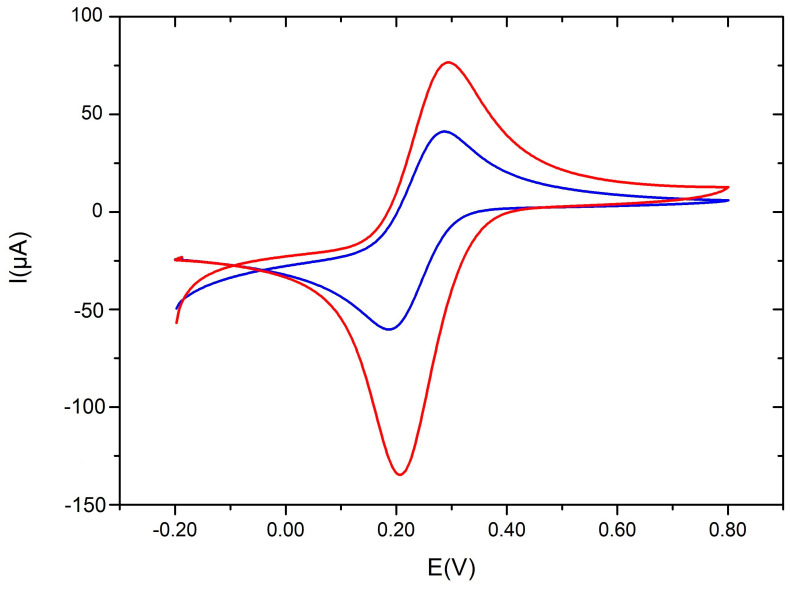
Cyclic voltammetry of 1.00 × 10^−2^ mol L^−1^ K_3_[Fe(CN)_6_] recorded at bare electrode (blue) and at CDs-CS/GCE-modified electrode (red); scan rate 0.020 V s^−1^; reference electrode Ag/AgCl.

Moreover, a quasi-reversible process was observed, based on the ratio anodic peak current/cathodic peak current (*I*_ap_/*I*_cp_), ranging from 0.81 to 1.33, and the difference between the anodic peak potential and the cathodic one (ΔE_p_), ranging from 0.22 to 0.08 V [35]. A more efficient electron transfer was generally observed for CDs-CS-modified electrodes with respect to the corresponding bare electrode, CDs-CS/GCE providing the best response, as shown in Figure 2.

### 3.3. Electrochemical Behavior of CAF and THEO at CDs-CS-Modified Electrodes

The anodic oxidation of CAF and THEO at solid electrodes in different medium had been previously investigated [5,11,36,37]. 

In the present paper, the electrochemical behavior of CAF and THEO was studied at bare and CDs-CS-modified electrodes to evaluate the effect of modifying the electrode surface.

The anodic peak potential (E_ap_) values appeared not significantly affected by the modification. Conversely, the electroanalytical improvement concerning the higher anodic peak current at the modified electrodes (data of CAF and THEO in Table 2 and Table 3, respectively) was evident, which was likely due to the increased sensing surface, the improved electrical connectivity network, and the higher chemical accessibility of the analyte through the CDs-CS network. The highest amplification of the peak current was achieved at the modified GCE. Moreover, the highest peak current amplification was observed for CAF, and this is not easy to explain considering the similar structure of the two molecules.

Therefore, the CDs-CS/GCE-modified electrode was chosen for further experiments for the simultaneous determination of CAF and THEO.

Successive measurements were carried out by differential pulse voltammetry (DPV), which was selected as a more sensitive voltammetric technique. DPV was used to investigate the effect of pH on the anodic oxidation and the effect of analyte concentration. Step potential, modulation amplitude, modulation time and scan rate were optimized as 0.004 V, 0.025 V, 0.05 s and 0.02 V s^−1^, respectively.

**Figure 3:**
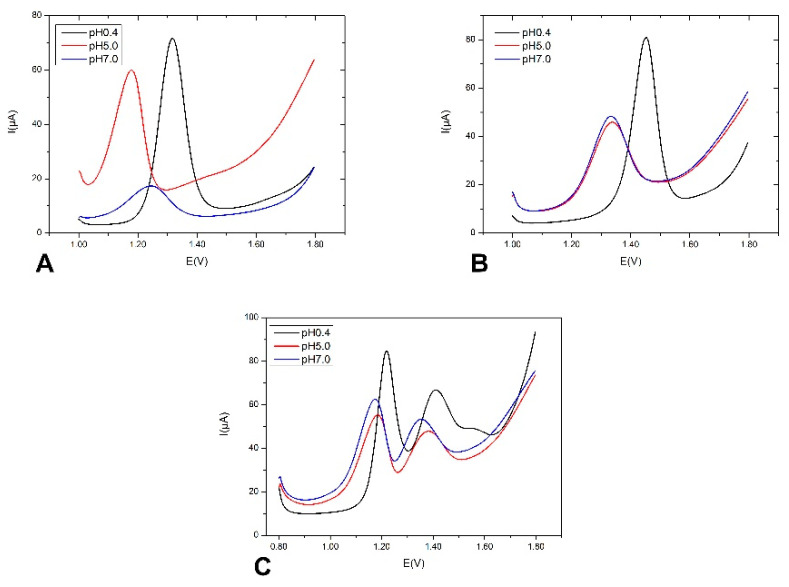
DPVs at CDs-CS/GCE at different pH values of: (**A**) THEO 5 × 10^−3^ mol L^−1^, (**B**) CAF 5 × 10^−3^ mol L^−1^, (**C**) CAF + THEO, both 5 × 10^−3^ mol L^−1^ in the ratio 1:1. Step potential: 0.004 V; modulation amplitude: 0.025 V; modulation time: 0.05 s; scan rate: 0.02 V s^−1^. Black: pH 0.4; red: pH 5; blue: pH 7.

Within the pH range 0.4–7, the E_p_ values of CAF (+1.49 V, Table 2) and THEO (+1.25 V, Table 3) decreased about 0.1 V for increasing pH, indicating that protons are involved in the oxidation mechanism. DPVs at CDs-CS-GCE of THEO 5 × 10^−3^ mol L^−1^ (A), CAF 5 × 10^−3^ mol L^−1^ (B) and CAF + THEO, both 5 × 10^−3^ mol L^−1^ in the ratio 1:1 (C), at three different pH values, namely 0.4, 5 and 7, are shown in Figure 3.

Moreover, the peak current increased for decreasing pH for both CAF and THEO, reaching a maximum at pH 0.4. A further peak current decrease was observed at higher pH values. Consequently, pH = 0.4 was selected for the detection of CAF and THEO.

### 3.4. Analysis of CAF and THEO by DPV at CDs-CS-GCE

The analysis of CAF and THEO was thus carried out by DPV at the best-performing modified electrode CDs-CS-GCE in aqueous medium containing HClO_4_ 0.4 mol L^−1^. 

The calibration curves were obtained individually for CAF and THEO by reporting the mean value of six consecutive DPV measurements for nine concentration points in the range 1 × 10^−5^–5 × 10^−3^ mol L^−1^. Simultaneous analysis was also performed, maintaining for each concentration point the ratio CAF/THEO, 1:1. Analogous results were obtained.

**Figure 4:**
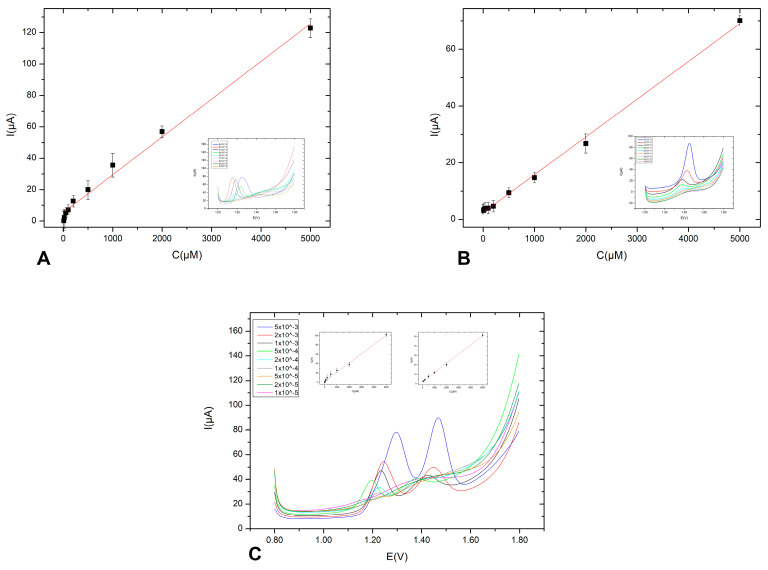
Calibration curves of (**A**) THEO, (**B**) CAF and DPV profiles of (**C**) THEO + CAF in the concentration ratio 1:1, in 0.4 mol L^−1^ HClO_4_, concentrations range 1 × 10^−5^–5 × 10^−3^ mol L^−1^, at CDs-CS-GCE, by DPV measurements (step potential 0.004 V, modulation amplitude 0.025 V, modulation time 0.05 s, scan rate 0.02 V s^−1^, vs. Ag/AgCl).

A very good linearity was found for both the analytes in the investigated range as supported by the R^2^ value, which was 0.997 and 0.990 for CAF and THEO, respectively. Linear equations, R^2^ values and limit of detection (LOD) are resumed in Table 4. Calibration curves of CAF and THEO and DPV profiles of CAF and THEO at different concentrations, in the ratio 1:1, are shown in Figure 4.

The effect of possible interfering compounds was evaluated at both bare and modified CDs-CS-GCE.

It has long been known that CAF interacts with polyphenolic molecules in aqueous solution [22], which is the reason why ferulic acid (FA), 5-caffeoyilquinic acid (CQA), catechin (C) and epicatechin (EC), usually found in tea containing CAF and THEO, were tested.

DPV voltammograms were recorded in 0.4 mol L^−1^ HClO_4_ containing CAF or THEO, in turn, 5.0 × 10^−3^ mol L^−1^, and CAF and THEO (concentration ratio, 1:1), in the absence and in the presence of the interfering compound.

No significant interaction was observed, as evidenced by the results shown in Table 5. 

The repeatability of DPV measurements was tested by constructing 10 successive calibration plots for CAF and THEO, individually, and for CAF and THEO in the same solution (concentration ratio, 1:1) with the same sensor. Relative standard deviation (RSD) values of 3.2%, 2.8% and 4.5% for CAF, THEO and for CAF and THEO (concentration ratio, 1:1), respectively, indicated a good repeatability with no need to apply any cleaning or regeneration procedure. 

The reproducibility of the sensor response was evaluated for seven electrodes (*n* = 7), using 2.0 × 10^−5^ mol L^−1^ solution of CAF and THEO, in turn, and CAF and THEO in the same solution (concentration ratio, 1:1), obtaining RSD values of 3.7%, 2.7% and 3.6%, respectively, suggesting a reliable construction procedure of the sensor.

The storage stability of the sensors was evaluated at +4 °C under wet conditions as well as at room temperature (RT) under dry conditions. The response of five sensors (*n* = 5) stored at +4 °C under wet conditions to 5.0 × 10^−4^ mol L^−1^ CAF and THEO, in turn, and CAF and THEO in the same solution (concentration ratio, 1:1), was tested every 3 days for 30 days; the average response decreased 15%, 20% and 22% after 30 days. The response of the sensors stored at RT in dry conditions was tested every 3 days for 30 days. After this period, the average response showed a decrease of almost 50% for CAF and THEO both individually and in the same solution (concentration ratio, 1:1).

### 3.5. Simultaneous Determination of CAF and THEO by HPLC-ESI-MS in SIR Mode

An independent analytical method based on chromatographic separation with a mass spectrometry detector for the simultaneous identification and quantitation of CAF and THEO in real samples was optimized, as reported in Section 2.6, and results were compared with those obtained by DPV at CDs-CS/GCE.

The Selected Ion Recording (SIR) technique was used as a highly selective and sensitive method for the analysis of real samples [23].

Calibration curves for CAF and THEO, obtained as described in Section 2.6, were constructed in the range 50–1000 μg L^−1^, at the concentrations of 50, 100, 300, 500, 700 and 1000 μg L^−1^. Linear equations, R^2^ values, LOD and LOQ for CAF and THEO are reported in Table 6.

### 3.6. Analysis of Real Samples 

The optimized CDs-CS/GCE sensor was applied for the simultaneous quantitation of CAF and THEO in real samples.

Three commercial teas in filter bags, namely Black Tea Darjeeling Coop (S1), Twinings Agrumance Tea (S2) and Everton Decaffeinated Earl Grey Green Tea (S3), prepared as described in Section 2.2, were analyzed. 

Due to the low amount of THEO found in the analyzed tea samples with respect to CAF, the optimized sensor was applied also for the determination of THEO in drug formulation, namely THEO-DUR 300 mg (S4), which was used as a bronchodilator for the treatment of bronchial asthma, and the sample was prepared as shown in Section 2.2. S4 is the only drug formulation available in Italy to date.

Finally, all the real samples were analyzed with the independent HPLC-ESI-MS in the SIR mode method optimized on purpose, and the results obtained in both experiments were compared.

The amounts of CAF and THEO found in the analyzed samples are reported in Table 7 as mean values ± standard deviation (SD) of three repeated DPV measurements at CDs-CS/GCE, and mean values ± SD of injection in triplicate by HPLC-ESI-MS in SIR mode.

Satisfactory results were obtained with CDs-CS/GCE for the quantitation of CAF in tea, as shown in Table 7. In particular, similar amounts of CAF were found by CDs-CS/GCE in both S1 and S2 samples, 1.40 × 10^−4^ and 1.60 × 10^−4^ mol L^−1^, respectively, whose results were not significantly different (*p* < 0.05, see Table 7, S1 and S2 samples, uppercase letter A). The same data were in good agreement with those ones obtained by HPLC-ESI-MS in SIR mode, 1.58 × 10^−4^ and 1.24 × 10^−4^ mol L^−1^ for S1 and S2 samples, respectively. In particular, the amounts of CAF in S1 were not significantly different when quantitated by CDs-CS-GCE and HPLC-ESI-MS (*p* < 0.05, see Table 7, S1 sample, lowercase letter a). 

Moreover, CAF was not detected by the electrochemical sensor in the declared decaffeinated tea S3, although a very low amount of CAF (6.06 × 10^−6^ mol L^−1^) was found in S3 by HPLC-ESI-MS: this result is consistent with the low LOQ values that can be achieved with a mass method with respect to electrochemical ones. 

Analogously, THEO was not detected by CDs-CS/GCE in any tea samples, S1, S2 and S3, although it was found in traces in the same samples by HPLC-ESI-MS. Nevertheless, these results can be considered consistent. In fact, the tea samples were very low in THEO: amounts under LOQ (9.39 × 10^−7^ mol L^−1^) were detected by HPLC-ESI-MS, which is the reason why the absence of detectable signals might be expected when the CDs-CS/GCE was used. For this reason, sample S4 was chosen to test CDs-CS/GCE for the quantitation of a higher content of THEO, as in a drug formulation as a real sample.

Less satisfactory results were obtained for the detection of THEO in drug, regarding either the amount and divergence occurring between the electrochemical and the HPLC-ESI-MS measurements.

According to the content declared by the manufacturer (300 mg/tablet), and based on the procedure described in Section 2.2. to prepare S4, the expected amount of THEO was 5.55 × 10^−3^ mol L^−1^, but both the CDs-CS/GCE and HPLC-ESI-MS method measured lower contents, 1.10 × 10^−3^ and 4.13 × 10^−3^ mol L^−1^, respectively (see Table 7): approximately −80% and −26%, respectively. The amounts of THEO in S4 quantitated by CDs-CS-GCE and HPLC-ESI-MS were significantly different (*p* < 0.05, see Table 7, S4 sample, lowercase letters a,b) when statistically analyzed, too. 

Such a discrepancy by both the methods suggested a matrix effect, which might be expected due to the excipients present in the drug and extracted by water along with THEO. In particular, the presence of molecules or macromolecules with important steric hindrance might strongly limit the diffusion of THEO to the electrode surface, causing the stronger negative matrix effect.

**Figure 5:**
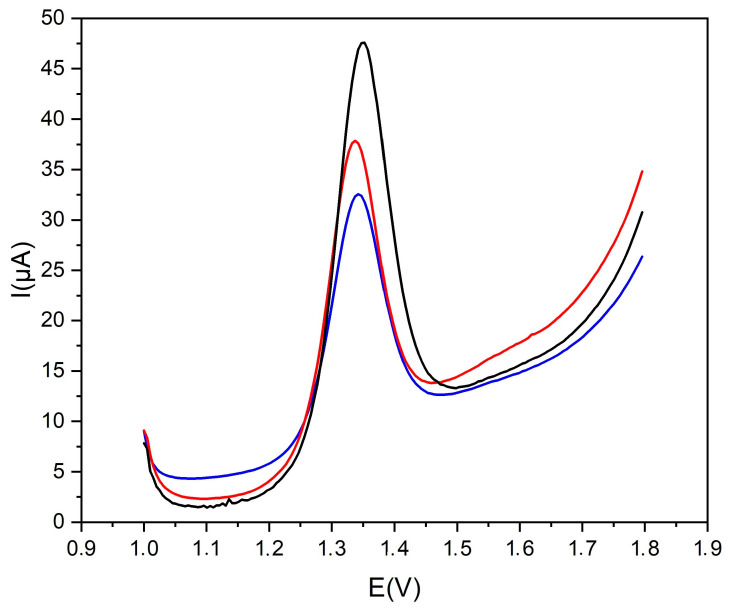
DPV voltammograms of THEO in drug samples recorded at CDs-CS/GCE: drug sample (blue line); drug sample + THEO 1.00 × 10^−3^ mol L^−1^ (red line); drug sample + 4.00 × 10^−3^ mol L^−1^ (black line).

The matrix effect (ME) was evaluated by adding known concentrations of THEO standard solution (1 × 10^−3^ mol L^−1^ and 4 × 10^−3^ mol L^−1^) to sample S4. DPVs recorded at CDs-CS/GCE on the starting drug solution and after the addition of known amounts of THEO are shown in Figure 5.

Recoveries within the range of 30–50% evidenced that the quantitation of THEO by the proposed sensor was strongly affected by the matrix, which was probably due to the presence of sterically bulky excipients such as sucrose, lactose, hydroxypropylcellulose, glyceryl monostearate or acetophthalate cellulose. They are soluble or partially soluble in water, not electrochemically active, and can be assumed as multifunctional excipients fulfilling multiple roles in a dosage form or drug delivery system, for example acting as filler material and at the same time as a binder and/or disintegrant [38]. On the other hand, they may slow or even hinder the diffusion of the analyte to the electrode. Finally, as THEO-DUR is a controlled-release drug, excipients play a very important role, acting as polymeric membranes in tablets with a multi-particulate formulation [38].

For completeness, the matrix effect (ME) for the quantitation of THEO in drug solution analyzed by HPLC-ESI-MS was evaluated as reported in the literature [26], constructing a calibration curve in the matrix by spiking three increasing amounts of THEO. ME of −67.79% was obtained, confirming a strong matrix effect for THEO in drug solution [39]. Conversely, the ME values for both CAF and THEO evaluated in the tea sample S2 were found to be −33% and −30%, respectively, confirming a weak to a weakly medium ME [39] in the less complex tea matrix. Although the ME in tea was not evaluated by the CDs-CS-GCE method, the good agreement of data with HPLC-ESI-MS ones suggests a similar behavior. 

The results obtained by the developed CDs-CS-GCE sensor were compared with data in the literature. Several examples of electrochemical sensors have been reported for the quantitation of CAF and THEO. The most common analytical parameters, such as linearity range, LOD and application media of a series of literature data were compared and resumed in Table 8 [40,41,42,43,44,45,46,47,48,49,50,51,52,53,54,55,56,57,58,59,60,61,62,63,64,65]. Most of the electrochemical sensors showed a limited linearity range, generally two or three orders of magnitude, confining the field of application to specific matrices, e.g., only teas. LOD values appeared in many cases in the range 10^−8^–10^−6^ mol L^−1^. A lower LOD of 3.3 × 10^−9^ mol L^−1^ was reported for THEO; nevertheless, a limited linearity range (1.00 × 10^−8^–1.00 × 10^−6^ mol L^−1^) makes the sensor not applicable to different matrices.

In most cases, no comparison with an independent method was reported.

## 4. Conclusions

In the present work, a glassy carbon electrode (GCE) modified with a composite including carbon dots (CDs) electrochemically synthesized and chitosan (CS) has been developed for the simultaneous quantitation of caffeine and theophylline in acidic medium by differential pulse voltammetry. To our knowledge, no previous studies on CDs-modified GCE for the analysis of these natural methylxantines have been reported.

The CDs-CS-GCE sensor was characterized by a three orders of magnitude linearity range and a limit of detection suitable for CAF and THEO in tea, and as a result, it performed in terms of storage stability, reproducibility and response similarly to some common interfering compounds.

Applied for the quantitation of CAF and THEO in real samples of tea, it provided results in very good agreement with those obtained by an independent HPLC-ESI/MS method optimized on purpose. Some differences in sensitivity were obviously evidenced, which were due to the known highly performing mass spectrometry methods. On the other hand, the electrochemical sensor presents undoubtable advantages in terms of cheapness, portability, and ease of use, since it does not require sophisticated equipment and skilled personnel.

## Figures and Tables

**Table 1:** Electrochemical data of 1.00 × 10^−2^ mol L^−1^ K_3_[Fe(CN)_6_], used as redox probe, by cyclic voltammetry, scan rate 0.020 V s^−1^, at bare electrodes (GCE, SPCE 110 and SPCE 150) and the corresponding best-performing modified electrode, by drop casting (CDs-CS/GCE ^b^) and electrodeposition (CDs-CS/SPCE 110 ^c^ and CDs-CS/SPCE 150 ^c^).

Electrode	E_ap_ (V)	E_cp_ (V)	*I*_ap_ (μA)	*I*_cp_ (μA)	*I*_ap_/*I*_cp_	ΔE_p_ (V)	Δ*I*_ap_ (%) ^a^	Area (cm^2^)
Bare GCE	0.28	0.19	56.82	53.86	1.05	0.09	-	2.20 × 10^−2^
CDs-CS/GCE ^b^	0.29	0.21	86.73	107.60	0.81	0.08	52.63	3.40 × 10^−2^
Bare SPCE 110	0.23	0.01	69.92	49.97	1.33	0.22	-	2.71 × 10^−2^
CDs-CS/SPCE 110 ^c^	0.17	0.06	89.98	69.46	1.30	0.11	28.70	3.51 × 10^−2^
Bare SPCE 150	0.23	0.03	68.58	57.30	1.20	0.20	-	2.70 × 10^−2^
CDs-CS/SPCE 150 ^c^	0.18	0.06	86.92	67.81	1.30	0.12	26.90	3.41 × 10^−2^

^a^: Δ*I*_ap_ was calculated using the equation (*I*_apM_ − (*I*_apB_/*I*_apB_)) × 100, where *I*_ap*M*_ and *I*_apB_ are the anodic current intensities at modified and bare electrodes, respectively; ^b^: drop-casting modification; ^c^: electrodeposition modification.

**Table 2:** Cyclic voltammetry (CV) data of CAF in 0.4 mol L^−1^ HClO_4_ at bare and modified electrodes. CAF = 5.0 × 10^−3^ mol L^−1^, scan rate 0.020 V s^−1^, vs. Ag/AgCl reference electrode.

Electrode	E_ap_ (V)	ΔE_ap_ (V)	*I*_ap_ (μA)	Δ*I*_ap_ (%) ^a^
Bare GCE	1.47	-	48.85	-
CDs-CS/GCE ^b^	1.49	0.02	73.22	50.00
Bare SPCE 110	1.23	-	71.24	-
CDs-CS/SPCE 110 ^c^	1.15	−0.13	98.01	37.60
Bare SPCE 150	1.22	-	72.69	-
CDs-CS/SPCE 150 ^c^	1.20	−0.02	99.12	36.40

^a^: Δ*I*_ap_ was calculated using the equation (*I*_apM_ − (*I*_apB_/*I*_apB_)) × 100, where *I*_ap*M*_ and *I*_apB_ are the anodic current intensities at modified and bare electrodes, respectively; ^b^: drop-casting modification; ^c^: electrodeposition modification.

**Table 3:** Cyclic voltammetry (CV) data of THEO in 0.4 mol L^−1^ HClO_4_ at bare and modified electrodes. THEO = 5.0 × 10^−3^ mol L^−1^, scan rate 0.020 V s^−1^, vs. Ag/AgCl reference electrode.

Electrode	E_ap_ (V)	ΔE_ap_ (V)	*I*_ap_ (μA)	Δ*Ι*_ap_ (%) ^a^
Bare GCE	1.26	-	94.74	-
CDs-CS/GCE ^b^	1.25	−0.01	119.63	26.30
Bare SPCE 110	1.02	-	96.18	-
CDs-CS/SPCE 110 ^c^	0.99	−0.10	106.22	10.43
Bare SPCE 150	1.09	-	105.67	-
CDs-CS/SPCE 150 ^c^	1.06	−0.03	112.81	6.80

^a^: Δ*I*_ap_ was calculated using the equation (*I*_apM_ − (*I*_apB_/*I*_apB_)) × 100, where *I*_ap*M*_ and *I*_apB_ are the anodic current intensities at modified and bare electrodes, respectively; ^b^: drop-casting modification; ^c^: electrodeposition modification.

**Table 4:** Linearity parameters and LOD (S/N = 3) for CAF and THEO, in the concentration range 1 × 10^−5^–5 × 10^−3^ mol L^−1^, by DPV at CDs-CS-GCE, in 0.4 mol L^−1^ HClO_4_.

Analyte	Linear Equation	R^2^	LOD
CAF	*I*_ap_ = 2.45 + 13,312.61 c	0.997	1 × 10^−6^ mol L^−1^
THEO	*I*_ap_ = 5.45 + 24,051.37 c	0.990	1 × 10^−6^ mol L^−1^

**Table 5:** DPV response of CAF and THEO at CDs-CS/GCE in the absence (no interferent) and in the presence of interfering compounds; FA: ferulic acid; CQA: 5-caffeoylquinic acid; C: catechin; EC: epicatechin.

Analyte	DPV Response (μA)				
	No Interferent	FA	CQA	C	EC
**CAF**	65.47	64.22	64.45	64.34	65.31
**THEO**	69.73	68.14	69.22	69.10	69.34

**Table 6:** Linearity parameters, LOD and LOQ for CAF and THEO, in the concentration range 50–1000 μg L^−1^, six points, by HPLC-ESI-MS in SIR mode, positive ionization (ES+).

Analyte	Linear Equation	R^2^	LOD	LOQ
CAF	y = 2287.65x + 15,910.55	0.9991	43.24 μg L^−1^ 2.23 × 10^−7^ mol L^−1^	144.12 μg L^−1^ 7.43 × 10^−7^ mol L^−1^
THEO	y = 2007.84x + 4644.84	0.9995	58.83 μg L^−1^ 3.27 × 10^−7^ mol L^−1^	169.09 μg L^−1^ 9.39 × 10^−7^ mol L^−1^

**Table 7:** Amounts (mol L^−1^ ± SD as mean values of analysis in triplicate) of CAF and THEO in three real samples of tea, S1 (Black Tea Darjeeeling Coop), S2 (Agrumance Twinings Tea), S3 (Everton Decaffeinated Earl Grey Green Tea), and one drug sample, S4 (THEO-DUR 300 mg), quantitated by CDs-CS/GCE (samples diluted with 0.4 mol L^−1^ HClO_4_) and by HPLC-ESI-MS in SIR mode (samples appropriately diluted in the mobile phase, A:B, 95:5, *v*:*v*).

Matrix	Analyte	CDs-CS/GCE (mol L^−1^ ± SD)	HPLC-ESI-MS (mol L^−1^ ± SD)
S1	CAF	1.40 × 10^−4^ ± 2.15 × 10^−5 a,A^	1.58 × 10^−4^ ± 7.58 × 10^−6 a,A^
S2	CAF	1.60 × 10^−4^ ± 7.63 × 10^−6 a,A^	1.24 × 10^−4^ ± 2.42 × 10^−6 b,B^
S3	CAF	nd	6.06 × 10^−6^ ± 1.84 × 10^−7^
S1	THEO	nd	<LOQ
S2	THEO	nd	<LOQ
S3	THEO	nd	<LOQ
S4	THEO	1.10 × 10^−3^ ± 1.56 × 10^−4 a^	4.13 × 10^−3^ ± 1.00 × 10^−4 b^

nd: not detected; <LOQ: under limit of quantitation (9.39 × 10^−7^ mol L^−1^); ^a,b^ refer to the same analyte (CAF or THEO) quantitated in the same sample (S1, S2, or S4) by different technique (CDs-CS-GCE and HPLC-ESI-MS); ^A,B^ refer to the same analyte (CAF or THEO) quantitated in different samples (S1, S2, S4) by the same technique (CDs-CS-GCE or HPLC-ESI-MS).

**Table 8:** An overview of electroanalytical methods for CAF and THEO determination.

Electrode	Analyte	Technique	Linearity (mol L^−1^)	LOD (mol L^−1^)	Application	Ref.
AuNPs-CS/AuE	CAF	DPV	2.00 × 10^−6^–5.00 × 10^−2^	1.00 × 10^−6^	Cola beverages, energy drink, teas	[22]
BiF/SPCE	CAF	DPV	1.00 × 10^−7^–2.00 × 10^−5^	2.7 × 10^−8^	River water samples	[40]
G/SPCE	CAF	DPAdSV	1.00 × 10^−7^–9.00 × 10^−7^ 1.00 × 10^−6^–1.00 × 10^−5^	2.0 × 10^−9^	Beverages, coffee	[41]
PDA/AuNPs/GCE	CAF	DPV	up to 7.50 × 10^−3^	7.90 × 10^−6^	Tea samples	[42]
MWCNTs/AuE	CAF	DPV	5.8 × 10^−7–^1.15 × 10^−5^	8.00 × 10^−8^	Tea samples	[43]
ZMWCNTsMCPE/SDS	CAF	SWV	1.0 × 10^−5^–1.0 × 10^−4^ 1.0 × 10^−4^–5.0 × 10^−4^	7.5 × 10^−8^	Black tea, coffee, Nescafe’, Pepsi Cola, energy drink, pharmaceutical tablets	[44]
MIP-TiO_2_ NPs	CAF	DPV	5.00 × 10^−6^–1.20 × 10^−4^	6.00 × 10^−6^	Green tea	[45]
Pt-G/GCE	CAF	DPV	9.00 × 10^−5^–3.80 × 10^−4^	1.13 × 10^−7^	Teas	[46]
NCoMCPE	CAF	DPV	5.00 × 10^−6^–6.00 × 10^−4^	1.60 × 10^−8^	Energy drink, pharmaceutical tablets	[47]
GrRACPE	CAF	DPV	5.00 × 10^−6^–1.00 × 10^−3^	2.90 × 10^−6^	Pepsi Cola, Coca Cola, energy drink, pharmaceutical tablets	[48]
GrRGCPE	CAF	DPV	5.00 × 10^−6^–1.00 × 10^−3^	6.10 × 10^−6^	Pepsi Cola, Coca Cola, energy drink, pharmaceutical tablets	[48]
Co_3_O_4_/GCE-Nafion	CAF	SWV	5.00 × 10^−6^–2.00 × 10^−4^	2.30 × 10^−7^	Teas	[49]
Co_3_O_4_/GCE-Nafion	CAF	Amperometry	4.00 × 10^−6^–5.00 × 10^−4^	9.70 × 10^−8^	Teas	[49]
bPGE	CAF	DPV	3.00 × 10^−5^–1.00 × 10^−3^	8.84 × 10^−6^	Cola, energy drink	[50]
bPGE	CAF	SWV	1.00 × 10^−5^–1.00 × 10^−3^	9.36 × 10^−6^	Cola, energy drink	[50]
AuNPs@PPy NTs/GCE	CAF	DPV	5.00 × 10^−8^–5.00 × 10^−4^	1.42 × 10^−8^	Coffee, energy drinks	[51]
AuNPs@PPy CNTs/GCE	CAF	DPV	1.00 × 10^−8^–1.00 × 10^−5^	2.80 × 10^−9^	Coffee, energy drinks	[51]
CNFs/GCE	CAF	SWV	2.50 × 10^−5^–4.50 × 10^−4^	1.74 × 10^−5^	Pharmaceutical tablets	[52]
WO_3_/MWCNT/GCE	THEO	DPAdSV	2.50 × 10^−8^–2.60 × 10^−6^	8.00 × 10^−9^	Clinical samples, pharmaceutical tablets	[53]
TiO_2_ MPs@GOs/GCE	THEO	Amperometry	2.00 × 10^−8^–2.10 × 10^−4^	1.32 × 10^−8^	Clinical samples, pharmaceutical tablets	[54]
GO-NC/GCE	THEO	SWV	3.00 × 10^−8^–5.00 × 10^−4^	3.12 × 10^−9^	Clinical samples, pharmaceutical tablets	[55]
CDs/GCE	THEO	DPV	1.00 × 10^−5^–2.00 × 10^−4^	3.30 × 10^−6^	Clinical samples	[56]
CuO-NPs/CPE	THEO	ALSV	4.00 × 10^−9^–7.00 × 10^−8^	1.20 × 10^−9^	Pharmaceutical tablets	[56]
CuO-GO/CPE	THEO	DPV	1.00 × 10^−7^–3.5 × 10^−6^	8.33 × 10^−9^	Clinical samples	[58]
Ni-Co MOF/GCE	THEO	DPV	1.00 × 10^−8^–1.00 × 10^−6^	3.3 × 10^−9^	Black tea	[59]
DP-Py COF/AuNPs/GCE	CAF + THEO	DPV	CAF 3.0 × 10^−5^–6.00 × 10^−4^ THEO 9.00 × 10^−5^–2.00 × 10^−5^ 2.00 × 10^−7^–4.00 × 10^−4^	CAF 1.90 × 10^−7^ THEO 7.60 × 10^−8^	Black tea, pharmaceutical tablets	[60]
MoS_2/_PANI@g-C_3_N_4_/ GCE	CAF + THEO	DPV	CAF 9.8 × 10^−6^–1.04 × 10^−4^ THEO 6.6 × 10^−6^–9.8 × 10^−5^	CAF 6.10 × 10^−8^ THEO 5.20 × 10^−8^	Ice cream, THEO tablets, energy drinks	[62]
βH-MnO2-NF/GCE	CAF + THEO	DPV	CAF 1.00 × 10^−8^–3.20 × 10^−4^ THEO 1.00 × 10^−8^–3.20 × 10^−4^	CAF 1.01 × 10^−8^ THEO 5.9 × 10^−9^	Chocolate, black tea, coffee powder, THEO tablet	[63]
MoS_2_/PANI/f-MWCNTs/GCE	CAF + THEO	DPV	CAF 5.3 × 10^−6^–1.25×10^−4^ THEO 5.3 × 10^−6^–1.25×10^−4^	CAF 5.10 × 10^−8^ THEO 4.20 × 10^−8^	Biscuits, THEO tablets, energy drinks	[64]
Ca_2_CuO_3_/GCE	CAF + THEO	DPV	CAF 1.75 × 10^−6^–2.11×10^−3^ THEO 2.50 × 10^−7^–2.07×10^−3^	CAF 1.72 × 10^−6^ THEO 1.05 × 10^−7^	Clinical samples	[65]
CDs/GCE	CAF + THEO	DPV	CAF 1 × 10^−5^–5 × 10^−3^ THEO 1 × 10^−5^–5 × 10^−3^ mol L^−1^	CAF 1.00 × 10^−6^ THEO 1.00 × 10^−6^	Teas, drugs	This work

## Data Availability

Not applicable.
